# A novel *PLEC* nonsense homozygous mutation (c.7159G > T; p.Glu2387*) causes epidermolysis bullosa simplex with muscular dystrophy and diffuse alopecia: a case report

**DOI:** 10.1186/s12895-018-0069-x

**Published:** 2018-01-20

**Authors:** Zoe Argyropoulou, Lu Liu, Linda Ozoemena, Claudia C. Branco, Raquel Senra, Ângela Reis-Rego, Luisa Mota-Vieira

**Affiliations:** 1 Molecular Genetics and Pathology Unit, Hospital of Divino Espírito Santo of Ponta Delgada, EPER, Av. D. Manuel I, 9500-370, Ponta Delgada, São Miguel Island, Azores Portugal; 2 The National Diagnostic EB Lab, St Thomas’ Hospital, London, UK; 3 BioISI – Biosystems & Integrative Sciences Institute, Faculty of Sciences, University of Lisboa, Lisbon, Portugal; 4 Instituto Gulbenkian de Ciência, Oeiras, Portugal; 5 Internal Medicine Department, Hospital of Divino Espírito Santo of Ponta Delgada, EPER, Ponta Delgada, Azores Islands Portugal

**Keywords:** Epidermolysis bullosa, Alopecia, Clinical dermatology, Azores

## Abstract

**Background:**

Epidermolysis bullosa simplex with muscular dystrophy (EBS-MD; OMIM #226670) is an autosomal recessive disease, characterized mainly by skin blistering at birth or shortly thereafter, progressive muscle weakness, and rarely by alopecia. EBS-MD is caused by mutations in the *PLEC* gene (OMIM *601282), which encodes plectin, a structural protein expressed in several tissues, including epithelia and muscle. We describe a patient affected with EBS-MD and diffuse alopecia in which we identified a novel pathogenic mutation by PCR amplification of all coding exons and exon–intron boundaries of *PLEC* gene, followed by bidirectional Sanger sequencing.

**Case presentation:**

The patient, a 28-year-old female and only child of consanguineous healthy parents, was born after uneventful pregnancy. At 2 days of age, she developed skin and oral mucosal blistering, accompanied by voice hoarseness. On physical examination as an adult, we observed diffuse non-scarring alopecia on the scalp, onychodystrophy (pachyonychia) in all 20 nails, dental decay, mild dysphonia, and severe muscle atrophy mainly affecting the extremities. Neurological examination showed profoundly diminished reflexes. Mutation analysis revealed the patient to be homozygous for the novel *PLEC* nonsense mutation − c.7159G > T (p.Glu2387*) − located in exon 31. Thismutation predicts the lack of expression of the full-length plectin isoform.

**Conclusion:**

The present case appears to be the second association of EBS-MD with diffuse alopecia, both cases having different mutations involving *PLEC* exon 31. It remains to be elucidated whether diffuse alopecia results from *PLEC* mutations and/or from environmental factors.

## Background

The name epidermolysis bullosa (EB) was introduced in 1886 and refers to a group of mechanobullous genodermatoses, defined by varying degrees of skin fragility caused by mutations in different skin structural proteins. Conventionally, four major types have been identified: EB simplex (EBS), junctional EB (JEB), dystrophic EB (DEB) and Kindler syndrome, each encompassing several subtypes [1, 2]. Epidermolysis bullosa simplex with muscular dystrophy (EBS-MD; OMIM #226670) is an autosomal recessive disease, characterized mainly by skin blistering at birth or shortly thereafter (the primary clinical manifestation) and by progressive muscle weakness with highly variable age of onset (from infancy to the fourth decade of life). Other clinical features include congenital onychodystrophy, mucous membrane involvement, and systemic symptoms (e.g., abnormal dentition, laryngeal webs, respiratory complications, and urethral strictures) [3]. This disease is caused by mutations in the *PLEC* gene (OMIM *601282), which encodes the cytolinker protein plectin expressed in many tissues, including the skin and muscle [3, 4]. The human *PLEC* gene presents, so far, eight alternative first exons, encoding different tissue-specific plectin isoforms [5]. For example, in mouse studies, transcripts containing exon 1d were exclusively found in skeletal and heart muscle, whereas exon 1a containing transcripts were dominant in organs rich in epithelial cell types, such as lung, small intestine, and, in particular, skin [5]. Plectin is a member of the plakin family of proteins, and plays a critical role in the formation of hemidesmosomes and securing the interactions of intermediate filaments to the plasma membrane attachment sites [6].

During the Fourth International Consensus Meeting on Diagnosis and Classification of EB [2], new definitions were discussed and published [1, 2]. In this revision, scalp abnormalities were not listed as a core clinical feature in EBS, including EBS-MD. Here, we describe a patient affected with EBS-MD and diffuse alopecia, where we identified a novel pathogenic mutation, in homozygous state, in the *PLEC* gene.

## Case presentation

**Fig. 1:**
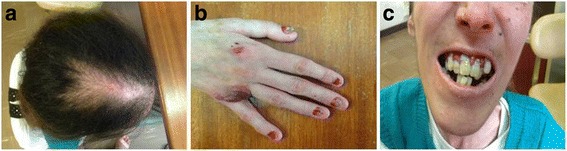
Clinical features in the 28-year-old female with epidermolysis bullosa simplex with muscular dystrophy and diffuse alopecia. This patient is originated from the Azorean island of São Miguel (Portugal). **a** Diffuse alopecia of the scalp, (**b**) Sparse and hemorrhagic blistering of the hand and onychodystrophy, and (**c**) Oral cavity abnormalities, such as caries and enamel hypoplasia

The patient is a 28-year-old female and the only child of consanguineous (first degree cousins, *F* = 0.0625) healthy parents from the Azorean island of São Miguel (Portugal), born after an uneventful full-term pregnancy (Table 1). From the second day after birth, she developed hemorrhagic blistering of the skin and oral mucosa, accompanied by a hoarse cry. In addition, at an early age (around 5–6 years old), she suffered from recurrent upper tract respiratory infections, and showed extensive nail dystrophy and hair weakness. No testing of the morphologic features of the hair fiber and shaft was performed; however the hair was thin and dull with extensive shedding. All developmental milestones were normal, with exception of weight (below the 5th percentile). The skin blistering gradually diminished with age, while hair loss from the scalp and eyebrows, without preceding blistering in those areas, progressively increased. Muscle weakness was first noted in adolescence and gradually progressed, resulting in an inability to perform activities of daily living. No similar cases were detected in the family. On physical examination, we observed sparse, tense hemorrhagic blisters and crusts distributed over the patient’s body. Diffuse non-scarring alopecia was evident on the scalp (Fig. 1a) and rarefaction of the eyebrows. Laboratory findings, such as thyroid hormone function, showed no hormonal alterations, and there is no familial history of alopecia. Onychodystrophy (pachyonychia) was found to affect all 20 nails (Fig. 1b). She also had dental abnormalities (caries and enamel hypoplasia; Fig. 1c), dysphonia, and severe muscle atrophy in the extremities. On the neurological examination, reflexes were profoundly diminished.

**Table 1:** Clinical description of the present case and its comparison with patients affected with EBS-MD and alopecia

Epidermolysis bullosa simplex with muscular dystrophy				
	Diffuse alopecia		Partial scarring alopecia	
Features	Present case	Yin J et al. [10]	Shimizu H et al. [11]	Shimizu H et al. [11]
*PLEC* gene mutations				
-status	Homozygous	Compound heterozygous	Compound heterozygous	Compound heterozygous
-exons	31	31	24 and 31	31 and 32
-DNA and protein level	c.7159G > T (p.Gln2387*)	c.4924C > T (p.Gln1642*)	c.3157C > T (p.Gln1053*)	c.7261C > T (p.Arg2421*)
	c.7159G > T (p.Gln2387*)	c.6955C > T (p.Arg2319*)	c.5806C > T (p.Gln1936*)	c.12578_12581dup (p.Tyr4195fs)
General clinical data				
Age (years)	28	25	9	33
Gender	F	F	M	M
Consanguinity	Present	Present	Present	Absent
Pregnancy	Full term/ Uneventful	Full term/ Uneventful	NA	NA
Skin involvement				
-skin blistering age onset	Neonatal	Neonatal	Birth	Neonatal
-skin blistering evolution	Diminished with age	Diminished with age	NA	Augmented every summer
-nail	Present	Present	Present	Present
-teeth	Present	Present	Present	Present
-focal plantar keratodermy	Absent	Absent	Absent	Absent
Muscle involvement				
-age onset	Adolescence	Adolescence	Infancy	Infancy
-evolution	Muscle atrophy 28y unable to perform activities of daily living	Muscle atrophy 20y unable to walk long distances or climb stairs	Major difficulties in walking at 9y	Muscle atrophy 20y unable to walk
Mucosa involvement				
-oral mucosal blistering	Present	Absent	Present	Present
-hoarsness	Present	Present (adolescence)	NA	NA
-laryngeal web	ND	NA	NA	NA
-urethral stricture	Absent	NA	Present	NA
Recurrent infections				
Upper respiratory tract (childhood)	Present	NA	NA	NA

*NA* Not available, *ND* Not determined

**Fig. 2:**
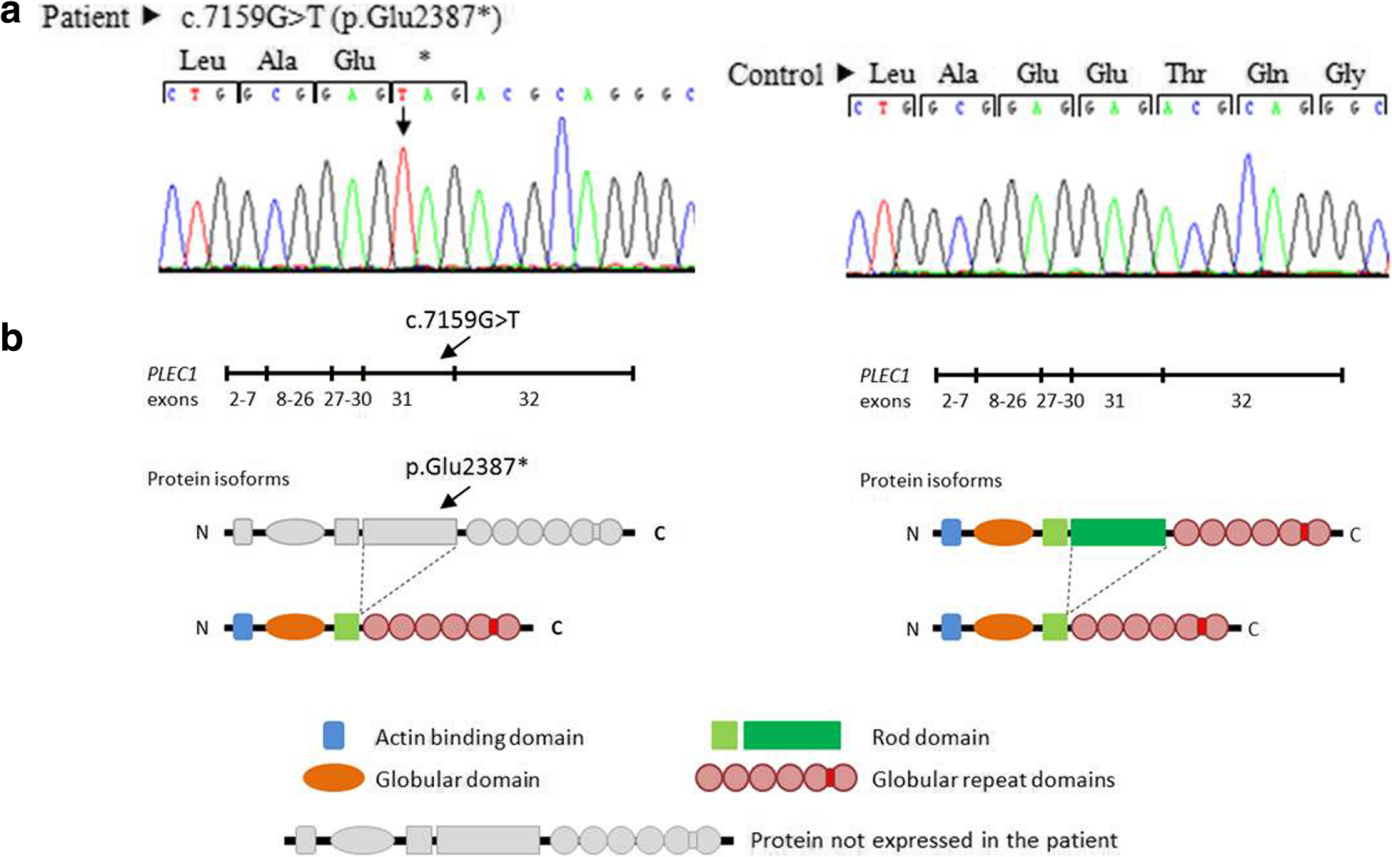
Mutation analysis of the *PLEC* gene. **a** Sequence chromatogram on genomic DNA from the patient and from an unaffected individual (control). Black arrow shows the novel nonsense mutation: c.7159G > T (p.Glu2387*) in exon 31, according to the isoform 1c: NM_000445/NP_000336. **b** Schematic representation of plectin polypeptides presumably present in the patient (rodless truncated isoforms)

To identify the pathogenic mutation, we performed PCR amplification of all coding exons and exon–intron boundaries of the *PLEC* gene (isoforms 1a, 1b, and 1c), followed by bidirectional Sanger sequencing, after obtaining the patient’s informed consent (the study was conducted according to the Declaration of Helsinki Principles). This strategy revealed the patient to be homozygous for the novel nonsense mutation *PLEC* (NM_000445):c.7159G > T (NP_000336:p.Glu2387*) in exon 31 (according to the isoform 1c; Fig. 2a). Bioinformatic analysis, by the ConSeq server (https://conseq.tau.ac.il/), revealed a score of 9 at this mutated position, indicating a very high sequence evolutionary conservation (data not shown). In order to predict whether the mutation has an impact on the protein biological function, we used PROVEAN (http://provean.jcvi.org/seq_submit.php) and Mutation Taster (http://www.mutationtaster.org/) tools. Both results showed a probable deleterious effect with a PROVEAN score of − 3.158 (cut-off value of − 2.5), and a prediction value of 1 for Mutation Taster (a value close to 1 indicates a high “security” of the prediction). The *PLEC*:c.7159G > T (p.Glu2387*) mutation results in a premature stop codon, which predicts truncated polypeptides and may also cause down regulation of the corresponding mRNA through nonsense-mediated mRNA decay (not evaluated in present case). Putative splice site variants were also analyzed by two in silico tools: NNSPLICE version 0.9 (http://www.fruitfly.org/seq_tools/splice.html) and Human Splicing Finder (http://www.umd.be/HSF3/), both suggesting that this mutation has no impact on splicing, i.e., there are no evidences of creating new acceptor and donor splicing sites.

## Discussion and conclusions

The protein plectin is a multimodular cytolinker of the plakin family, which interacts with intermediate filaments and anchors them at strategic sites for cells organization and performance, including peripheral cell junctions, intracellular structures, and organelles [7, 8]. In addition, plectin interacts with other cytoskeletal elements, such as actin and microtubules. There are two major isoforms of plectin: full-length and rodless plectin; the latter lacks the rod domain due to alternative splicing of transcripts that lack exon 31 [7, 8]. Here, we identified a novel *PLEC* homozygous mutation – c.7159G > T (p.Glu2387*) – in an EBS-MD patient with diffuse alopecia. The bioinformatic analysis of this nonsense mutation, located in exon 31, suggests that it is the disease causing variant, probably by the lack of plectin full-length variant and/or expression of the rodless isoform (Fig. 2b). Previous studies, performed by Sawamura et al. [9], indicate that exon 31 alternative splicing may restore the *PLEC* open-reading frame in some EBS-MD patients, allowing a partial rescue of the phenotype. This may explain the mild clinical features observed in the patient, including the relatively late onset muscular dystrophy. Another explanation may be the difference in the amount of full-length and rodless plectin between skin and muscle, as observed in Natsuga work [4]. This work estimated that the ratio of full-length to rodless transcripts is 10:1 in skin, although, in human skeletal muscle, it is possible that the amount of both isoforms is comparable [4]. Taken together, these observations elucidate the temporal difference in symptoms appearance, this is, skin fragility is typically found in infancy, and muscular dystrophy onset is relatively delayed.

According to the recommendations on diagnosis and classification of EB [1, 2], diffuse alopecia, present in junctional epidermolysis bullosa (JEB) generalized intermediate subtype, is a very uncommon clinical feature associated with EBS-MD. To our knowledge, the present case corresponds to the second one of EBS-MD and diffuse alopecia, described in the literature, with a novel mutation in *PLEC* gene [c.7159G > T (p.Glu2387*)]. The other case is a compound heterozygous for two different mutations in exon 31 [c.4924C > T (p.Gln1642*) and c.6955C > T (p.Arg2319*); Table 1] [10]. On the other hand, EBS-MD and partial scarring alopecia was observed in only two patients [11]. Both were compound heterozygous for a mutation on exon 31 and mutations on exons 24 or 32: one patient [c.3157C > T (p.Gln1053*); c.5806C > T (p.Gln1936*)]; and the other [c.7261C > T (p.Arg2421*); (c.12578_12581dup (p.Tyr4195fs)]. We hypothesise the involvement of *PLEC* exon 31 in alopecia, although our suggestion is only based on these four cases. Even though we can rule out hormonal imbalance and familial history, it remains to be elucidated whether diffuse alopecia results from *PLEC* mutations and/or a combination of other factors, such as stress and nutrition. In conclusion, we report a novel mutation leading to truncated plectin in an Azorean patient with EBS–MD and diffuse alopecia.

## Abbreviations

DEB: Dystrophic epidermolysis bullosa
EB: Epidermolysis bullosa
EBS: Epidermolysis bullosa simplex
EBS-MD: Epidermolysis bullosa simplex with muscular dystrophy
JEB: Junctional epidermolysis bullosa
